# Sweet Chestnut (*Castanea sativa* Mill.) Bark Extract: Cardiovascular Activity and Myocyte Protection against Oxidative Damage

**DOI:** 10.1155/2013/471790

**Published:** 2013-02-27

**Authors:** Alberto Chiarini, Matteo Micucci, Marco Malaguti, Roberta Budriesi, Pierfranco Ioan, Monia Lenzi, Carmela Fimognari, Tullia Gallina Toschi, Patrizia Comandini, Silvana Hrelia

**Affiliations:** ^1^Department of Pharmacy and Biotechnology, University of Bologna, Via Belmeloro 6, 40126 Bologna, Italy; ^2^Department of Life Quality Studies, University of Bologna, Via Irnerio 48, 40126 Bologna, Italy; ^3^Department of Agricultural and Food Sciences, University of Bologna, Piazza Goidanich, 60, 47521 Cesena, Italy

## Abstract

This work was aimed at evaluating the cardioprotective effects of *Castanea sativa* Mill. (CSM) bark extract characterized in its phenolic composition by HPLC-DAD-MS analysis. The study was performed using primary cultures of neonatal rat cardiomyocytes to investigate the antioxidant and cytoprotective effects of CSM bark extract and isolated guinea pig left and right atria, left papillary muscle, and aorta to evaluate its direct effect on cholinergic and adrenergic response. In cultured cardiomyocytes the CSM bark extract reduced intracellular reactive oxygen species formation and improved cell viability following oxidative stress in dose-dependent manner. Moreover, the extract decreased the contraction induced by noradrenaline (1 **μ**M) in guinea pig aortic strips and induced transient negative chronotropic and positive inotropic effects without involvement of cholinergic or adrenergic receptors in the guinea pig atria. Our results indicate that CSM bark extract exhibits antioxidant activity and might induce cardioprotective effect.

## 1. Introduction

Many natural compounds have a wide range of biological activities including antioxidant, chemopreventive, anti-inflammatory, neuroprotective, and cardioprotective effects.

Sweet chestnut (*Castanea sativa* Mill.) is a known source of phenolic bioactive compounds, in particular of tannins [1]. Tannins have been classified into two majors groups: hydrolysable and condensed tannins [2]. Hydrolysable tannins can be subdivided into two subclasses: gallotannins and ellagitannins. Gallotannins have a core of *β*-penta-O-galloyl-D-glucopyranose to which several other galloyl ester groups are linked despite fashion. Ellagitannins are characterized by the presence of two C–C-coupled galloyl ester groups at the 2,3-and/or 4,6-positions of a ^4^C1-glucopyranose [3].

The wide variety of structures is due to many different linkage possibilities between esters and glucose. Gallotannins and ellagitannins are present in several species of *Fagaceae* family [4] such as oak and chestnut [1]. Different studies indicate that ellagitannins have antiatherogenic, anti-thrombotic, anti-inflammatory, and antiangiogenic properties [5] and antioxidant activity [6], and the main ellagitannin metabolites, urolithins, exert a strong antioxidant activity [7].

Condensed tannins (proanthocyanidins) are oligomers or polymers of flavan-3-ol and contain no sugar residues [1]. Proanthocyanidines, classified in different subgroups, are important components of food and are abundant in red wine, green tea, and chocolate [8]. Proanthocyanidines, especially those obtained from grape seeds, have been widely studied for their ability to attenuate the cardiovascular, cerebrovascular, and peripheral vascular risk. Proanthocyanidines, together with resveratrol, have been recently demonstrated to be responsible for the cardioprotective effect of red wine [9, 10]. Cardioprotective effects of proanthocyanidines have been attributed to their strong antioxidant activity, to the blood vessel relaxing ability by acting on endothelium-dependent relaxing cells [11], to the inhibition of angiotensin-I converting enzyme [12], and to their antiarrhythmic effect able to decrease tissue injury induced by ischemia/reperfusion [13].

Sweet chestnut is a tree belonging to the *Fagaceae* family, present in Mediterranean regions of Europe. The decoction of its leaves has been widely used, in folk medicine, for treating various respiratory diseases such as asthma, cough, cold, bronchitis, expectorating, and bronchial affections. Calliste et al. described CSM leaves as a source of natural antioxidants [14]. Recently, a natural extract from the bark of CSM has been shown to exert antispasmodic action induced by modulation of cholinergic receptors and calcium channels [15]. In addition, the extract has also antiviral effect against different viruses [16]. Furthermore, sweet chestnut wood extract administration reduced oxidative stress induced by high n-3 polyunsaturated fatty acid (n-3 PUFA) intake in young pigs and the formation of toxic products of PUFA oxidation; in addition, it prevented DNA damage in blood lymphocytes [17].

To our knowledge, no data are available to elucidate the effects of CSM bark extracts on cardiac cells, and no data about their cardioprotective effects are reported in the literature. In this study, we have fully characterized CSM bark extract in its polyphenol composition, and we demonstrated its ability to act as antioxidant and cytoprotective agent in rat cultured cardiomyocytes. Moreover, the direct CSM bark extract effect on the cardiovascular system has been investigated, by the evaluation of inotropic and chronotropic effects on guinea pig isolated left and right atria and papillary muscle and its activity in guinea pig isolated aortic strips.

## 2. Materials and Methods

### 2.1. Materials

CSM bark extract was supplied and obtained as previously reported [15]. Methanol (p.a.), monohydrate gallic acid (99.1%) and ellagic acid (≥96%), 3-(4,5-dimethylthiazol-2-yl)-2,5-diphenyltetrazolium bromide (MTT), 2′,7′-dichlorodihydrofluorescein diacetate (DCFH-DA), and all other chemicals were purchased from Sigma-Aldrich (St. Louis, MO, USA). Acetonitrile (gradient grade, for HPLC) was from VWR (Milano, Italy), and formic acid (98%–100%) was from Merck (Darmstadt, Germany). Deionized water was obtained from an Elix 10 water purification system from Millipore (Bedford, MA, USA).

### 2.2. Spectrophotometric Determination of Total Phenol Content

The total phenol (TP) content of tannin extract was determined by adapting the method used by Pirisi et al. [18]. After the extraction with methanol and a suitable dilution of the sample, TP content was determined by using the Folin-Ciocalteu reagent and measuring the absorbance at 750 nm (Shimadzu Spectrophotometer UV-VIS 1204, Kyoto, Japan). TP content was calculated using gallic acid for the construction of the calibration curve (*r* ^2^ = 0.9967), expressing the results as g of gallic acid/100 g of dry extract, designated as gallic acid equivalent (GAE)/100 g.

### 2.3. HPLC-DAD-MS Analysis

The dry extract was dissolved in methanol and analysed in HPLC-DAD-MS (HP 1100 Series, Agilent Technologies, Palo Alto, CA, USA). The analysis has been realized on a C18 column (Phenomenex, Torrance, CA, USA; 5 *μ*m, 150 mm × 4.6 mm id). The mobile phase was composed of a solvent A: 0.5% v/v formic acid in HPLC-grade water and a solvent B: acetonitrile. An injection volume of 20 *μ*L and a flow rate of 0.5 mL/min were used. The detector wavelength was set at 254 nm. All the analyses were carried out at room temperature.

The following conditions of ESI interface were used: drying gas flow, 9.0 L/min; nebulizer pressure, 35 psig; gas drying temperature, 350°C; capillary voltage, 3000 V; fragmentor voltage, 60 V.

Phenolic and tannin compounds were identified comparing retention times and UV and MS spectra of the detected peaks with those of commercial standards (gallic and ellagic acids); if reference compounds were not available, a tentative identification was made by analyzing and comparing elution order and spectroscopic and spectrometric information with the literature data. The quantification of each compound was performed using eight-point regression curves obtained using gallic (*r* ^2^ = 0.999) or ellagic acid (*r* ^2^ = 0.999).

Tannins and other phenolic compounds were quantified as g of ellagic acid/100 g and g of gallic acid/100 g of dry extract and indicated, respectively, as ellagic acid equivalent (EAE)/100 g and gallic acid equivalent (GAE)/100 g.

### 2.4. Cell Culture and Treatments

Neonatal cardiac myocytes were isolated as previously reported [19]. The investigation conforms to the Guide for the Care and Use of Laboratory Animals published by the U.S. National Institutes of Health (NIH Publication 85-23, revised 1996) and approved by the Ethics Committee of our institution. Briefly, cells, obtained from the ventricles of 2–4-day-old rats, were grown until complete confluence. Cells were treated with different concentration of extract (1–500 *μ*g/mL) for 24 h, and control cells were treated with equivalent concentrations of DMSO alone.

### 2.5. Determination of Cell Viability

Cardiomyocyte viability of control and treated cells was measured using the MTT assay as previously reported [20]. For the flow cytometry analysis the cells were double-labelled with Annexin V conjugated to Phycoerythrin (Annexin V-PE) and 7-Amino-actinomycin D (7 AAD) and immediately analyzed on a Guava EasyCyte flow cytometer (Guava Technologies, Hayward, CA) in accordance with the manufacturer's instructions as reported in [21]. The percentage of viable cells was reported with respect to the total number of cells.

### 2.6. Detection of Intracellular Reactive Oxygen Species

The formation of reactive oxygen species (ROS) was evaluated using a fluorescent probe, DCFH-DA, as previously reported [22]. Briefly, controls and treated cells were washed with PBS and then incubated with 5 *μ*M DCFH-DA in PBS for 30 min. After DCFH-DA removal, the cells were incubated with 100 *μ*M H_2_O_2_ for 30 min. Cell fluorescence from each well was measured using a microplate spectrofluorometer (*λ*_ex_ = 485 nm and *λ*_em_ = 535 nm). Intracellular antioxidant activity was expressed as the percentage of inhibition of intracellular ROS produced by H_2_O_2_ exposure.

### 2.7. Determination of Cytoprotective Effect

Cytoprotection against H_2_O_2_-induced cell damage was assessed using the MTT assay as previously reported [20]. Control and treated cells were exposed to 100 *μ*M H_2_O_2_ in PBS for 30 min after which cells were changed to a fresh culture medium. After 24 h, MTT was added to the medium at the final concentration of 0.5 mg/mL and incubated for 1 h at 37°C. DMSO was added to dissolve the formazan crystals, and the absorbance was measured at 595 nm using a microplate reader VICTOR3 V Multilabel Counter. Data were expressed as percentage of viable cells with respect to control times.

### 2.8. Animals

All animals used in this study were housed and treated according to the directives on the protection of animals used for scientific purposes (Directive 2010/63/EU of the European Parliament and of the Council) and the WMA Statement on Animal Use in Biomedical Research. All procedures followed the guidelines of animal care and were approved by the Ethics Committee of the University of Bologna (Bologna, Italy). Briefly, guinea pigs (males and females, 300–400 g) obtained from Charles River (Calcio, Como, Italy) were housed in a controlled environment with a 12 : 12-h light-dark cycle at 22°C and provided with chow diet and water *ad libitum*.

### 2.9. Guinea Pig Atrial Preparations and Treatments

Experiments were set up as previously described [23]. Atrial muscle preparations were used to examine the inotropic and chronotropic activity of the CSM bark extract (0.01–10 mg/mL), dissolved in physiological salt solution (PSS). During the generation of cumulative concentration-response curves, the next higher concentration of extract was added only after the preparation reached a steady state. Some experiments were performed with a single extract concentration (1 mg/mL).

### 2.10. Muscarinic Activity

It was determined on guinea pig spontaneously beating right atria. After tissue stabilization, cumulative log concentration-response curves to the agonist Carbachol (CCh) (0.01–1 *μ*M) were constructed. Following incubation with the antagonist atropine (1 *μ*M) or by simultaneous administration of atropine (1 *μ*M) with extract (1 mg/mL) a new concentration-response curve to CCh was obtained. Parallel experiments in the absence of antagonist were run. One set of experiments was carried out using a single concentration of extract (1 mg/mL): in particular negative chronotropic activity was induced by a single dose of extract. Following incubation with atropine (1 *μ*M) for 30 min, a new effect with CSM bark extract (1 mg/mL) was done.

### 2.11. Adrenergic Activity

It was determined on guinea pig left atria driven at 1 Hz. After tissue stabilization, a contraction to Sweet Chestnut extract (1 mg/mL) was performed. Following incubation with propranolol (10^−6^ M) for 30 min, a new contraction to extract (1 mg/mL) was obtained. Simultaneously reproducibility of the contraction obtained by the first to the second trials in the absence of propranolol (10^−6^ M) was confirmed.

### 2.12. Guinea Pig Left Papillary Muscle Preparation

Experiments were set up as previously described [24]. Papillary muscle preparations were used to examine the inotropic activity of the CSM bark extract (0.01–10 mg/mL), dissolved in PSS. During the generation of cumulative concentration-response curves, the next higher concentration of extract was added only after the preparation reached a steady state. Some experiments were performed with a single extract concentration (1 mg/mL).

### 2.13. Guinea Pig Aortic Strips Preparation

Experiments were set up as previously described [24]. After the equilibration period, guinea pig aortic strips were contracted by washing in PSS containing 80 mM KCl (equimolar substitution of K^+^ for Na^+^) or 1 *μ*M noradrenaline (NA). When the contraction reached a plateau, different concentrations of the extract (0.01–10 mg/mL) were added cumulatively allowing any relaxation to obtain an equilibrated level of force. Some experiments were performed with a single extract concentration (1 mg/mL).

### 2.14. Statistical Analysis

Data obtained from rat neonatal cardiomyocytes cell cultures are presented as means ± S.D. and were analyzed by one-way analysis of variance (ANOVA) followed by Dunnett's test, and *P* value less than 0.05 has been considered significant. Data on atria, papillary muscle, and aortic strips were analyzed by the Student's *t-*test and presented as means ± S.E.M. [25]. *P* value less than 0.05 has been considered significant. The potency of drugs defined as EC_50_ was calculated from log concentration-response curves. Antagonist activity expressed as p*A*_2_ was calculated from Schild plots [26], constrained to slope −1.0 [25]. Three different antagonist concentrations were used, and each concentration was tested at least four times.

## 3. Results

### 3.1. Characterization of CSM Bark Extract

The TP content of CSM bark extract was firstly determined by Folin-Ciocalteu method [27].

**Figure 1:**
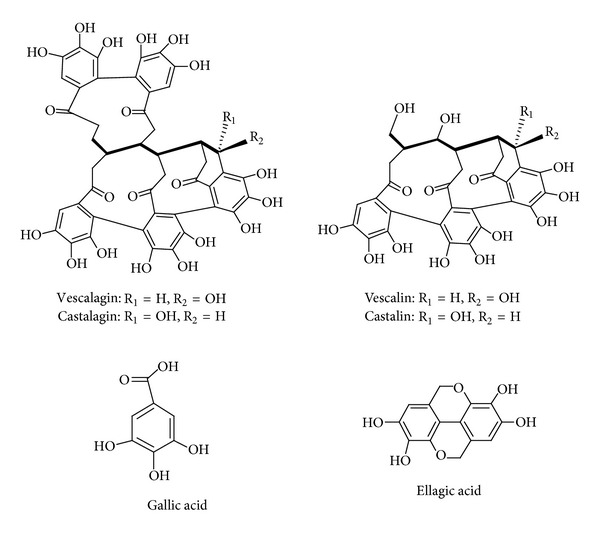
Chemical structure of detected compounds.

Based on the literature data [28–31], gallic acid was selected as reference standard for the TP content by Folin-Ciocalteu spectrophotometric method. TP content of the examined CSM bark extract was 54.9% of dry weight (g GAE/100 g of extract). A characterization of the extract was realized by HPLC. Tentative identification of tannins and phenolic compounds was made on the basis of retention time, molecular weight, spectroscopic properties, and MS fragmentation characteristics (ESI negative mode). The amount of tannins and phenolic compounds characterized in CSM bark extract, including ellagic acid, gallic acid, and 4 ellagitannins (vescalin, castalin, vescalagin and castalagin) (Figure 1), is reported in Table 1.

The three major components were vescalagin, castalagin, and ellagic acid. Other minor compounds, detected in trace levels, 5-*o*-galloylhamamelose, (3,5-dimethoxy-4-hydroxyphenol)-1-*o*-**β**-D-(6′-*o*-galloyl)-glucoside isomer, *m*-digallic acid, kurigalin isomer and chestanin, are reported as other compounds in Table 1.

The total amount of separated ellagitannins and phenols, expressed as ellagic acid, was 10.69 ± 0.28 g EAE/100 g, while it was more than double (24.01 ± 0.57) if expressed as gallic acid (g GAE/100 g).

### 3.2. Antioxidant and Cytoprotective Effects of CSM Bark Extract on Cultured Rat Cardiomyocytes

**Figure 2:**
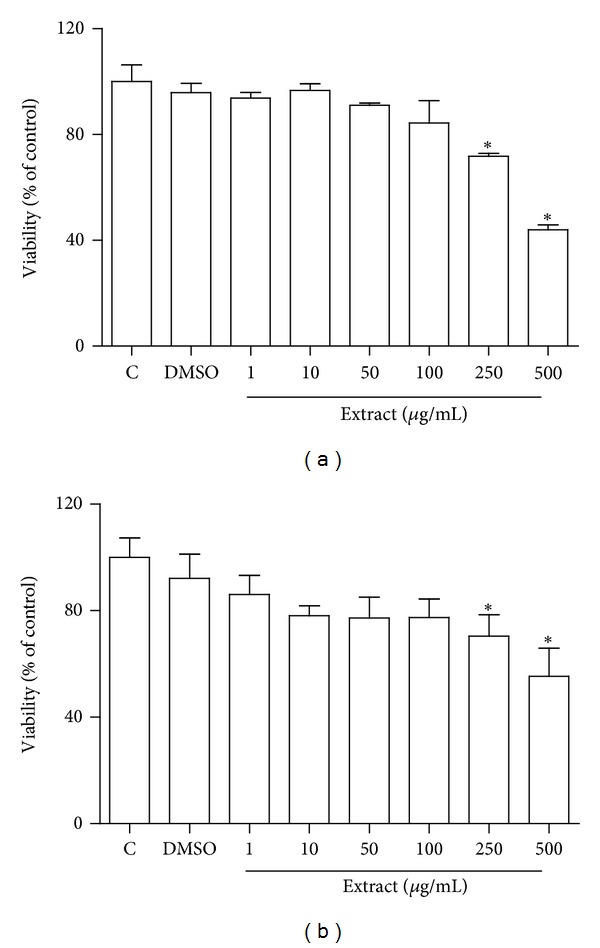
Cell viability of cultured cardiomyocytes treated with CSM bark extract. Rat cardiomyocytes were treated with CSM bark extract, solubilised in DMSO, as described in Materials and Methods section. (a) Cell viability was analysed by the MTT test as reported in Materials and Methods section, (b) cell viability was analysed by flow cytometry. Cells were double-labelled with Annexin V-PE 7 AAD and analyzed by a Guava EasyCyte flow cytometer. Data are reported as means ± S.D. *P < 0.05 with respect to controls.

Sweet chestnut bark extract is particularly rich in tannins; therefore, we have investigated the ability of the extract to protect cultured cardiomyocytes from oxidative stress.  Figure 2 shows, by both MTT (a) and flow cytometry analysis (b), that CSM extract did not exert any toxic effect on cultured primary cardiomyocytes on a wide range of concentrations (1–100 *μ*g/mL).

**Figure 3:**
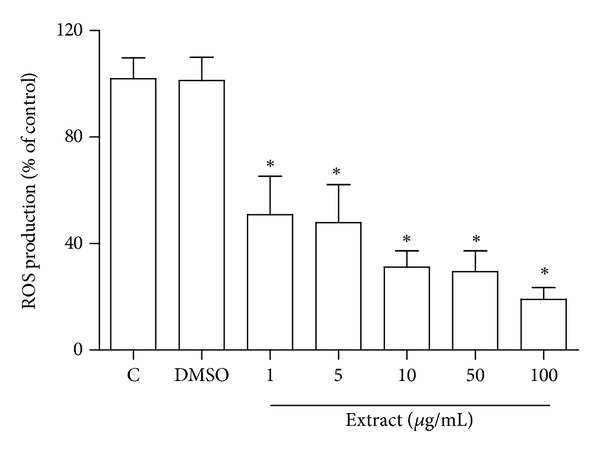
Effect of CSM bark extract on intracellular ROS production in cardiomyocytes. Cardiomyocytes were treated with chestnut extract (1–100 *μ*g/mL) for 24 h, oxidative damage was then induced exposing the cells to 100 *μ*M H_2_O_2_ for 30 min, and intracellular ROS were determined using the peroxide-sensitive fluorescent probe DCFH-DA as described in Materials and Methods section. Data are expressed as percent of control cells treated with H_2_O_2_. Values represent means ± S.D. (*n* = 4). *P < 0.05 with respect to H_2_O_2_-treated cells.

A significant decrease in ROS production, as detected by DCFH-DA assay, was observed in CSM bark extract-treated cardiomyocytes following exposure to H_2_O_2_ (Figure 3). The ability of the extract to reduce ROS production was already detected at 1 *μ*g/mL concentration.

Vehicle controls containing equivalent volumes of DMSO (0.2% v/v) did not show any significant difference in comparison to cells exposed to H_2_O_2_. ROS levels were significantly reduced in extract-treated cells after 24 h in a dose-dependent fashion.

**Figure 4:**
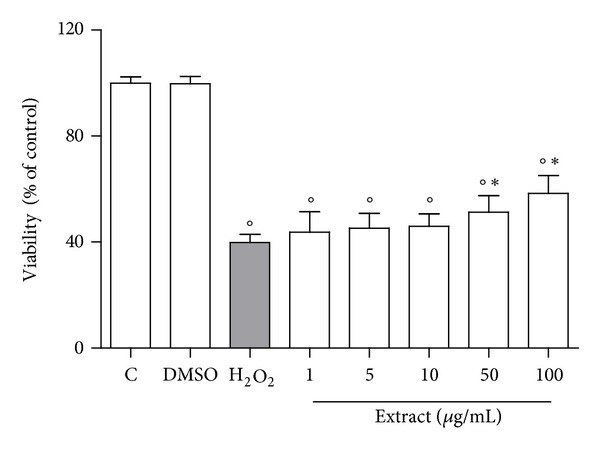
Effect of CSM bark extract treatment on cell viability in cardiomyocytes exposed to H_2_O_2_. Cardiomyocytes were treated with CSM bark extract (1–100 *μ*g/mL) for 24 h before the addition of 100 *μ*M H_2_O_2_, and cellular damage was assessed by the MTT assay and reported as percent cell viability in comparison to control cells. Each bar represents the mean ± S.D. of four independent experiments. *P < 0.05 with respect to H_2_O_2_-treated cells and °*P* < 0.05 with respect to control cells.

Incubation of cardiomyocytes with 100 *μ*M H_2_O_2_ for 30 min caused a significant decrease in cell viability (Figure 4), as detected by MTT reduction assay. Treatment of cardiac cells with CSM bark extract at the concentration of 50–100 *μ*g/mL for 24 h prior to H_2_O_2_ exposure partially protected against oxidative damage, as shown by the significant increase in cell viability with respect to H_2_O_2_-treated cells.

### 3.3. Effects of CSM Bark Extract on Guinea Pig Isolated Left and Right Atria and Papillary Muscle

CSM bark extract was tested in guinea pig left atrium and left papillary muscle driven at 1 Hz and in spontaneously beating right atrium to evaluate its inotropic and/or chronotropic effects, respectively. A single dose (1 mg/mL) of CSM bark extract produced a positive inotropic effect (*E*_max⁡_ = 218 ± 17%) in left atrium with a concomitant reduction in heart rate (*E*_max⁡_ = −59 ± 3.6%). The extract (1 mg/mL) revealed a positive inotropic effect also on the left papillary muscle stimulated at 1 Hz (*E*_max⁡_ = 42 ± 2.1%) (Table 2).

**Figure 5:**
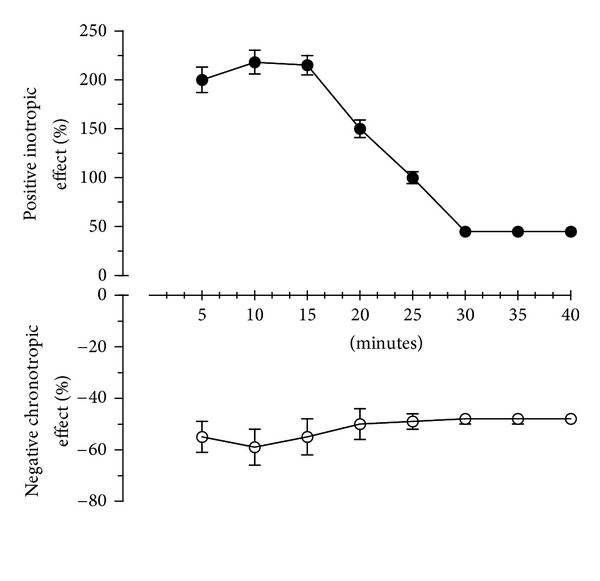
Time course of CSM bark extract treatment (1 mg/mL) on positive inotropic effect in guinea pig left atria driven at 1 Hz (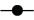) and on negative chronotropic effect in spontaneously beating right atria (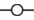), respectively. Values are means ± S.D. (*n* = 6).

All the reported effects were reversible after 30 minutes of washing. To evaluate the time course of simultaneous positive inotropic and negative chronotropic effects on left and right atria, respectively, we measured the effect of a single dose (1 mg/mL) every 5 minutes for 30 minutes (Figure 5).

The intrinsic positive inotropic activity was reduced by about 50% after 30 minutes (*E*_max⁡_ from 218 ± 17% to 45 ± 0.7%), whereas the negative chronotropic effect only slightly decreased at the same time (*E*_max⁡_ from −59 ± 3.6% to −48 ± 1.3%).

Data obtained by cumulative concentration-effect curves (data not shown) revealed that the intrinsic activity did not exceed 50% with the exception of positive inotropy on papillary muscle (*E*_max⁡_ = 52 ± 1.9%) (Table 3).

To clarify the mechanisms involved in the observed negative chronotropic effect and in order to elucidate the implication of cholinergic system, the spontaneously beating right atrium was treated with the extract in the presence and in the absence of 1 *μ*M atropine.

The presence of atropine did not modify the effect of the extract on heart rate or inotropy. Moreover, the p*A*_2_ of atropine did not change in the presence of the extract (Table 4), indicating that the chronotropic or inotropic effects are not mediated by cholinergic receptor modulation.

Since it has been previously shown that natural extract of chestnut is able to block gut M_3_ cholinergic receptors in a noncompetitive reversible manner [15], it is possible to hypothesize that the extract could discriminate between these two cholinergic receptor subtypes.

**Figure 6:**
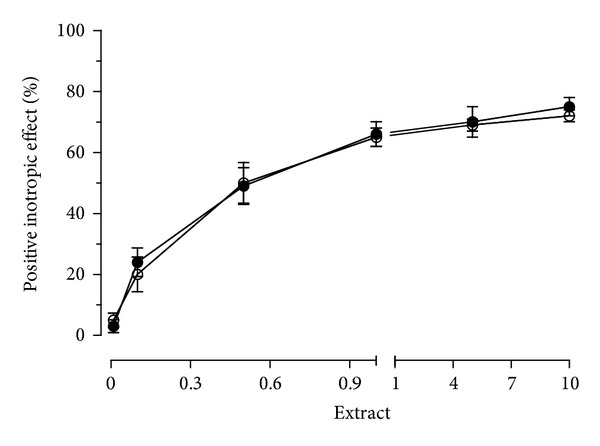
Cumulative concentration-response curve for CSM bark extract in the absence (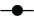) and in the presence of 1 *μ*M propranolol (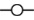) in guinea pig left atria driven at 1 Hz. Each point is the mean ± S.D. (*n* = 6).

As a positive inotropic effect was evidenced by CSM bark extract on guinea pig left atrium driven at 1 Hz, the involvement of adrenergic receptors was verified. The positive inotropic effect of CSM bark extract (1 mg/mL) in left atrium also persisted in the presence of 1 *μ*M propranolol (Figure 6).

### 3.4. Effects of CSM Bark Extract on Guinea Pig Aortic Strips

**Figure 7:**
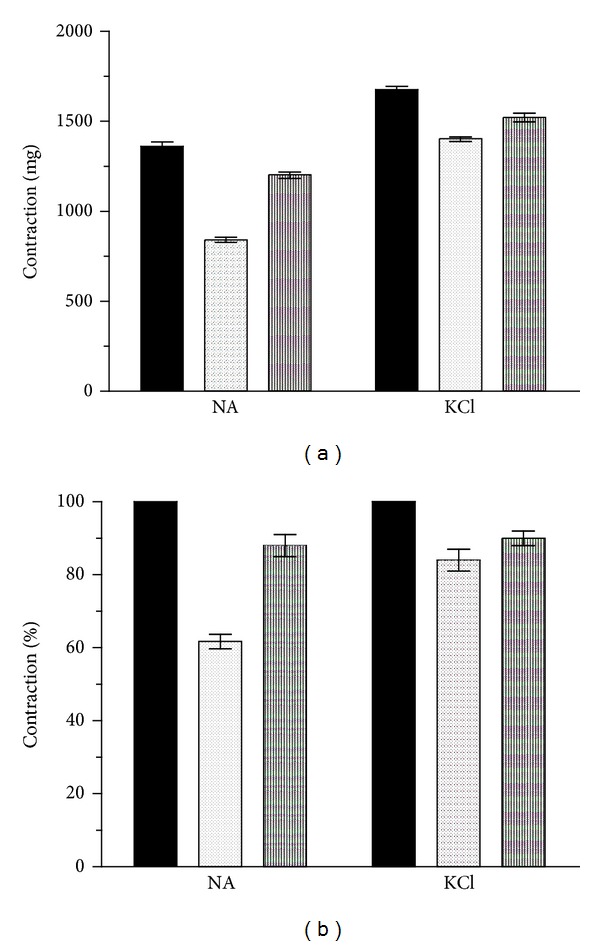
Spasmolytic activity of the CSM bark extract against contraction induced by 1 *μ*M NA or 80 mM KCl. (a) The effect was expressed in milligrams of contraction. (b) The effect was expressed as percentage of the control. Black bars represent the effect of agonist, dotted bars the effect of agonist in presence of chestnut extract (1 mg/mL), and ruled bars the effect of the agonist after washout of extract. Each point is the mean ± S.D. (*n* = 6).

CSM bark extract was tested on guinea pig aortic strips, in the presence of 80 mM KCl or 1 *μ*M NA, to assess the vasorelaxant activity. CSM bark extract (1 mg/mL) spasmolytic activity was about 50% against NA and less than 20% against KCl (Figure 7). After a washout of the extract the contraction induced by KCl and NA was similar to that induced by the agonist alone.

**Figure 8:**
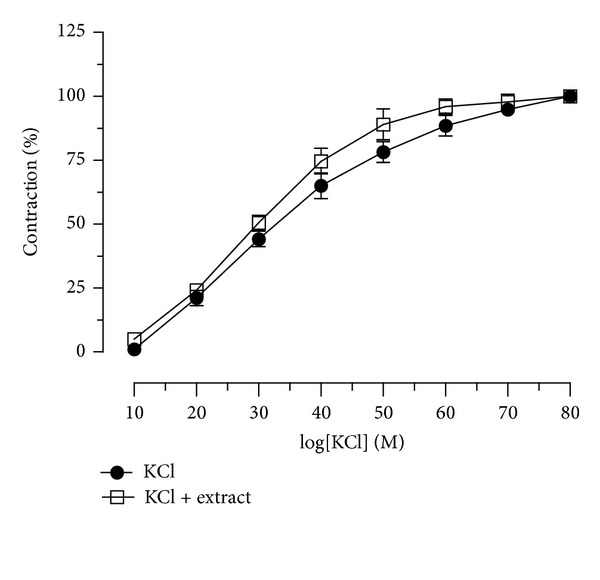
Effect of the extract on potassium chloride-induced contraction in isolated guinea pig aortic strips. Cumulative dose-response curves were obtained before and after exposure to the extract (1 mg/mL) for 30 min. Each point is the mean ± SEM (*n* = 5-6).

The spasmolytic activity of the extract against KCl-induced contraction was evaluated by cumulative dose-response curves. The curve of KCl contraction in the presence of CSM bark extract (1 mg/mL) was not significantly different from the control (Figure 8).

## 4. Discussion

Many studies described the relationships between cardiac dysfunction and initiating factors such as smoking, excessive alcohol consumption, diet, obesity, and stress. In several cases, the effect of one of these factors alone or in combination with others, as atherosclerosis and hypertension, represents a first step in the development of different cardiac dysfunctions such as angina, infarction, arrhythmias, or heart failure [32, 33] .

Different approaches are commonly applied to reduce cardiovascular risks. Beside the early identification of risk factors, lifestyles and nutrition habits play a fundamental role in preventing or counteracting cardiovascular diseases. Epidemiological studies have indicated the existence of an inverse correlation between the intake of fruits and vegetables, rich in antioxidant phytochemicals, and the risk of developing cardiovascular diseases [34, 35]. Many phytochemicals have been shown to exert antioxidant effect and to counteract many oxidative-stress-related diseases, like cardiovascular diseases [36, 37].

The bark and wood of chestnut trees are an important source of tannins. Different *Castanea* tissues are rich in both simple phenolics and tannins, and the chestnut wood contains much higher levels of phenolics than the chestnut fruits [38]. The extract used in this study has been demonstrated to contain more than 10% (w/w) of phenolic compounds, of which tannins as vescalagin and castalagin are the more representative. It has been previously demonstrated that many ellagitannins, including castalagin and vescalagin, have potent antitumor, antioxidant, antimicrobial and antimalarial, properties [6, 39–41].

Tannins, including proanthocyanidins, exert many biological effects [42]. Tannins exert a double-action towards the cardiovascular system: a direct action on heart and blood vessels by modulating cardiovascular parameters and an indirect action through their antioxidant activity. In fact they are able to inhibit lipid peroxidation and lipoxygenases in vitro and to scavenge radicals such as hydroxyl, superoxide, and peroxyl [43]. While antioxidant effects of condensed tannins have been reported by several studies [44], there is little information on the antioxidant activity of water soluble tannins [45, 46]. Hydrolysable tannins, for their high degree of hydroxylated aromatic functions, show high antioxidant activity [47].

In many countries Pycnogenol, a preparation based on *Pinus maritima* bark extract, is used as a cardioprotective food supplement [48]. The main bioactive compounds of this product are oligomeric proanthocyanidines and phenolics monomers.

Extracts from *Castanea Sativa* leaves have been shown to exert an antioxidant effect in different in vitro and in vivo model systems and to be useful in the prevention of photoaging and oxidative-stress-mediated skin diseases [14, 49].

Recently Frankic and Salobir [17] demonstrated a decreased level of many biomarkers of oxidative stress in pigs fed a chestnut wood extract supplemented diet and exposed to n-3 PUFA-induced oxidative stress. Moreover previous studies have demonstrated the ability of chestnut (*Castanea crenata*) inner shell extract to protect HepG2 cells from t-BHP-induced oxidative stress [50].

For the first time this paper reports the cytoprotective and antioxidant activity of CSM bark extract in cultured heart cells.

In this study CSM bark extract did not show any toxic effect in cultured cardiomyocytes in a wide range of concentrations (1 to 100 *μ*g/mL) and resulted in a significant protection against H_2_O_2_-induced cytotoxicity and in a marked decrease in intracellular ROS production. This is not surprising due to the high phenolic and tannin content of the extract.

Furthermore, ex vivo studies have been performed to better elucidate the cardioprotective effect of CSM bark extract using different tissue preparations.

In isolated atrial preparations, extract simultaneously induced transient negative chronotropic effect and positive inotropic effect. On papillary muscle, the positive inotropic effect is persistent and may be useful for ventricular support of heart function and for preventing stagnation of blood into the ventricles [51].

To our knowledge only few studies have investigated the cardiovascular effects of plant extracts. Ojewole et al. [52] demonstrated that the aqueous leaf extract of *Persea americana* Mill. exerted negative inotropic and negative chronotropic effects on guinea pig isolated electrically driven left and spontaneously beating right atrial muscle preparations, respectively. Moreover, *Persea americana* Mill. leaf extract reduced, in a concentration-dependent manner, the positive inotropic and chronotropic responses of guinea pig isolated atrial muscle strips induced by noradrenaline and calcium. According to their findings the authors concluded suggesting that *Persea americana* Mill. leaf could be used as a natural aid in essential hypertension.

Similarly Musabayane et al. [53] showed a concentration-dependent negative inotropic and chronotropic effect on rat isolated electrically driven left and spontaneously beating right atria preparations of *Helichrysum ceres* leaf ethanolic extract. The authors also demonstrated that *Helichrysum ceres* leaf ethanolic extract induces vasorelaxant effects on aortic strips preparations and that these effects are both mediated by NO-dependent and independent mechanisms.

In our study we have demonstrated that both chronotropic and inotropic effects of the extract are not due to the main receptor mechanisms involved in heart function regulation (i.e., muscarinic M_2_ and *β*-adrenergic receptors). These effects are shared, even though to a lesser extent, with digital, the well-known drug obtained from *Digitalis purpurea, *which exhibits both positive inotropic and negative chronotropic actions without involving any muscarinic and *β*-adrenergic receptor mechanisms.

Moreover, CSM bark extract did not significantly change the vascular contraction induced by 80 mM potassium or 1 *μ*M noradrenaline.

In summary data reported here demonstrate that the CSM bark extract has important antioxidant and cytoprotective effects and is also able to modulate some important cardiac functions. Notwithstanding the limitation derived by the use of different cell/tissue models, the results clearly indicate a nutraceutical value for the CSM bark extract and could suggest its use as nutritional supplement in the prevention of cardiovascular diseases.

Although further studies are needed to identify the biochemical and pharmacological mechanisms besides the positive inotropic and negative chronotropic effects exerted by the CSM bark extract, this natural extract could be a valuable support as dietary supplement, combining beneficial direct effects on cardiovascular functions with a high antioxidant activity.

## Figures and Tables

**Table 1:** Amounts of separated tannins and phenolic compounds in chestnut bark extract, expressed as g EAE /100 g and as g GAE/100 g.

Compound	g EAE/100 g^a^	g GAE/100 g^a^
Vescalin	0.56 ± 0.02	1.18 ± 0.06
Castalin	0.69 ± 0.02	1.47 ± 0.06
Gallic acid	1.25 ± 0.04	3.68 ± 0.12
Vescalagin	2.31 ± 0.05	5.01 ± 0.11
Castalagin	2.26 ± 0.07	4.96 ± 0.08
Ellagic acid	1.70 ± 0.05	3.64 ± 0.10
Other compounds	1.92 ± 0.04	4.07 ± 0.04

Total	10.69 ± 0.28	24.01 ± 0.57

^a^Values are means ± S.D. (*n* = 3).

**Table 2:** Single dose of sweet chestnut bark extract. Activity in guinea pig heart preparations.

Compound	Tissue		
	Left atrium	Right atrium	Papillary muscle
	Positive inotropic activity^a^	Negative chronotropic activity^b^	Positive inotropic activity^c^
Extract 1 mg/mL	218 ± 17	59 ± 3.6	42 ± 2.1

^a^Increase in developed tension on isolated guinea pig left atrium, expressed as percent changes from the controls (*n* = 5-6). The left atria were driven at 1 Hz.

^b^Decrease in atrial rate on guinea pig spontaneously beating isolated right atrium, expressed as percent changes from the control (*n* = 7-8). Pretreatment heart rate ranged from 165 to 190 beats/min.

^c^Increase in developed tension on isolated guinea pig left papillary muscle, expressed as percent changes from the control (*n* = 5-6). The left papillary muscle was driven at 1 Hz. Data represent mean ± S.E.M. All data refer to 10 minutes of incubation.

**Table 3:** Cumulative concentration-activity curve in guinea pig heart preparations. Cardiac activity and potency of sweet chestnut bark extract.

Compound	Positive inotropy				Negative chronotropy	
	Left atria		Left papillary muscle		Right atria	
	Activity^a^	Potency^b^	Activity^c^	Potency^b^	Activity^d^	Potency^b^
Extract	45 ± 0.7	—	52 ± 1.9	0.09 (c.l. 0.07–0.13)	48 ± 1.3	—

^a^Increase in developed tension on isolated guinea pig left atrium at 1 mg/mL extract concentration, expressed as percent changes from the control (*n* = 5-6). Data represent mean ± S.E.M. The left atria were driven at 1 Hz. 1 mg/mL extract concentration gave the maximum effect.

^b^The EC_50_ was expressed as mg/mL concentration that gave the 50% of the effect and was calculated from concentration-response curves (Probit analysis by Litchfield and Wilcoxon with *n* = 6-7).

^c^Increase in developed tension on isolated guinea pig left papillary muscle at 1 mg/mL extract concentration, expressed as percent changes from the control (*n* = 5-6). The left papillary muscle was driven at 1 Hz. 1 mg/mL gave the maximum effect for extract. Data represent mean ± S.E.M.

^d^Decrease in atrial rate on guinea pig spontaneously beating isolated right atrium at 1 mg/mL extract concentration, expressed as percent changes from the control (*n* = 7-8). Data represent mean ± S.E.M. Pretreatment heart rate ranged from 165 to 190 beats/min. 1 mg/mL gave the maximum effect for extract.

**Table 4:** Antagonist affinities at guinea pig right atria, expressed as p*A*_2_ values.

Compound	Right atrium p*A*_2_^a^	
	Inotropy	Chronotropy
Atropine	9.45 ± 0.04	9.21 ± 0.03
Atropine + extract	9.41 ± 0.03	9.19 ± 0.02

^a^The agonist was carbachol. Each p*A*_2_ value was obtained for three different concentrations and was calculated from Schild plots [25, 28]. Results are presented as mean ± S.E.M.
